# Pharmacologic Ascorbate and DNMT Inhibitors Increase DUOX Expression and Peroxide-Mediated Toxicity in Pancreatic Cancer

**DOI:** 10.3390/antiox12091683

**Published:** 2023-08-29

**Authors:** Garett J. Steers, Brianne R. O’Leary, Juan Du, Brett A. Wagner, Rory S. Carroll, Frederick E. Domann, Prabhat C. Goswami, Garry R. Buettner, Joseph J. Cullen

**Affiliations:** 1 Free Radical and Radiation Biology Division, Department of Radiation Oncology, Iowa City, IA 52242, USA; garett-steers@uiowa.edu (G.J.S.); brianne-oleary@uiowa.edu (B.R.O.); juan-du@uiowa.edu (J.D.); brett-wagner@uiowa.edu (B.A.W.); rory-carroll@uiowa.edu (R.S.C.); frederick-domann@uiowa.edu (F.E.D.); prabhat-goswami@uiowa.edu (P.C.G.); garry-buettner@uiowa.edu (G.R.B.); 2 The Department of Surgery, The University of Iowa Carver College of Medicine, Iowa City, IA 52242, USA

**Keywords:** ascorbic acid, pharmacologic ascorbate, pancreatic cancer, epigenetics, DNA methyltransferase (DNMT), ten-eleven translocation (TET) methylcytosine dioxygenase

## Abstract

Recent studies have demonstrated an important role for vitamin C in the epigenetic regulation of cancer-related genes via DNA demethylation by the ten-eleven translocation (TET) methylcytosine dioxygenase enzymes. DNA methyltransferase (DNMT) reverses this, increasing DNA methylation and decreasing gene expression. Dual oxidase (DUOX) enzymes produce hydrogen peroxide (H_2_O_2_) in normal pancreatic tissue but are silenced in pancreatic cancer (PDAC). Treatment of PDAC with pharmacologic ascorbate (P-AscH^−^, intravenous, high dose vitamin C) increases DUOX expression. We hypothesized that inhibiting DNMT may act synergistically with P-AscH^−^ to further increase DUOX expression and cytotoxicity of PDAC. PDAC cells demonstrated dose-dependent increases in DUOX mRNA and protein expression when treated with DNMT inhibitors. PDAC cells treated with P-AscH^−^ + DNMT inhibitors demonstrated increased DUOX expression, increased intracellular oxidation, and increased cytotoxicity in vitro and in vivo compared to either treatment alone. These findings suggest a potential therapeutic, epigenetic mechanism to treat PDAC.

## 1. Introduction

Pancreatic ductal adenocarcinoma (PDAC) currently accounts for approximately 3% of all new cancer diagnoses but for 8% of all cancer deaths, making it the third leading cause of cancer-related death in the United States [1]. The aggressive nature of PDAC is multifactorial and can be attributed to advanced stage at diagnosis and resistance to radiation and chemotherapy. Overall PDAC patients have a 5-year relative survival of about 11%; patients with metastatic disease have only a 3% 5-year relative survival, an especially grim prognosis considering nearly 50% of patients are found to have metastatic disease at their initial diagnosis [1]. Current chemotherapy regimens such as FOLFIRINOX (5-fluorouracil, oxaliplatin, irinotecan, and leucovorin) and gemcitabine plus nab-paclitaxel have offered modest improvements in survival [2]. These improvements have yielded median survival times of just 8–11 months in patients with metastatic disease and less than 2 years in patients with locally advanced disease [3,4]. Thus, new treatment modalities are critically important in improving survival among PDAC patients.

Pharmacologic ascorbate (P-AscH^−^, high-dose intravenous vitamin C) was first proposed as a cancer treatment in the 1970s but interest soon waned. However, it has experienced a resurgence in interest due to multiple clinical trials with encouraging results [5]. P-AscH^−^ acts as a pro-oxidant, delivering high concentrations of extracellular hydrogen peroxide (H_2_O_2_) to the cancer cell microenvironment [6]. H_2_O_2_ can easily cross the cell membrane and form the highly reactive hydroxyl radical (HO^•^), resulting in protein and DNA damage and ultimately cell death [6,7]. This H_2_O_2_-induced cytotoxicity has been shown to be selective to cancer cells due to their overall higher endogenous levels of reactive oxygen species (ROS) as well as lower levels of peroxide-removing enzymes such as catalase and glutathione peroxidase when compared to normal cells [6,8,9]. P-AscH^−^ has shown promise as an effective adjunct to standard-of-care chemotherapy in phase I and II clinical trials in PDAC, lung cancer, and glioblastoma in recent years [7,10,11,12].

In addition to its direct cytotoxic effects at pharmacologic doses, ascorbate has also been shown to act as an important cofactor for the activity of the ten-eleven translocation (TET) methylcytosine dioxygenase family of enzymes. These enzymes are responsible for removing methyl groups on cytosine bases in DNA, converting 5-methylcytosine (5-mC) to 5-hydroxymethylcytosine (5-hmC) [13,14]. Conversely, DNA methyltransferases (DNMT1, DNMT3A, DNMT3B) add methyl groups to cytosine bases [15,16]. The methylation status of 5′ cytosine bases within promoter regions and CpG islands influences gene expression and can therefore have a significant impact on the phenotype of tissues and organisms. Hypermethylation of promoter regions decreases downstream gene expression likely by altering chromatin organization and function as well as interaction with transcriptional repressors (methyl-CpG-binding proteins, MeCP1 and MeCP2) [16,17,18]. Hypermethylation within promoter regions of tumor suppressor and DNA repair genes has been identified in every major tumor type, including PDAC [19,20,21]. Relatedly, overexpression of DNMT has been associated with more aggressive forms of cancer, suggesting that DNMT overexpression leading to a hypermethylated state results in aberrant gene expression and decreased expression of tumor suppressor genes [22,23,24]. Studies have shown that PDAC is hypermethylated and exhibits DNMT1 overexpression, leading to reduced expression of many tumor suppressor genes including p16 and APC [20,25]. Previous studies have also demonstrated that DNMT1 inhibition in PDAC reduces cancer cell growth both in vitro and in vivo [26,27]. Inhibitors of DNMT such as 5-azacytidine and 5-aza-2′-deoxycytidine are frequently used in hematologic malignancies, such as myelodysplastic syndrome and acute myeloid leukemia, but their use is being increasingly investigated in solid organ tumors with promising results in early studies in colorectal cancer, ovarian cancer, and PDAC [25,28,29,30]. Reducing the degree of DNA methylation in PDAC may result in increased expression of epigenetically silenced tumor suppressor genes and help to enhance the cytotoxicity of other chemotherapeutic agents.

Two genes that are silenced in PDAC are dual oxidase 1 and 2 (DUOX 1 and 2), members of the NADPH oxidase family of enzymes that produce extracellular hydrogen peroxide (H_2_O_2_) in normal tissues throughout the body [31,32]. These genes have been shown to be epigenetically silenced in lung cancer, hepatocellular carcinoma, and PDAC, and their expression in PDAC is increased for up to 72 h following treatment with P-AscH^−^, leading to sustained oxidative stress and cytotoxicity [31,33,34]. We hypothesized that P-AscH^−^ and DNMT inhibitors may act synergistically to increase DUOX expression, leading to increased H_2_O_2_-induced cytotoxicity in PDAC. Here, we demonstrate that P-AscH^−^ and DNMT inhibitors act synergistically to reduce PDAC cell growth in a dose-dependent manner through an H_2_O_2_-dependent pathway secondary to increased DUOX expression. We also demonstrate the efficacy of P-AscH^−^ and DNMT inhibitors in vivo, where tumor volume was significantly reduced and DUOX1 expression was increased in a xenograft model. Combination treatment with P-AscH^−^ and DNMT inhibitors may offer an epigenetic approach to the treatment of PDAC.

## 2. Materials and Methods

Survival Curve Analysis—The NIH Genomic Data Commons (GDC) Cancer Genome Atlas (TCGA) Pancreatic Cancer Database (PAAD) (*n* = 223) was accessed via the University of California Santa Cruz (UCSC) Xena Functional Genomics Explorer [35]. The compared variables were “DNA methylation—Illumina Human Methylation 450” and “OS” (overall survival). The dataset was filtered using “sample type.samples”, “primary tumor”, “morphology.diagnoses”, and “8500/3” to only include primary tumor samples from patients with invasive ductal adenocarcinoma. Adenocarcinoma not otherwise specified (*n* = 24), neuroendocrine carcinoma (*n* = 9), mucinous adenocarcinoma (*n* = 6), adenocarcinoma with mixed subtypes (*n* = 1), and undifferentiated carcinoma (*n* = 1) were excluded. After excluding normal solid tissue (*n* = 37) and metastatic (*n* = 1) samples, the final number of samples for evaluation of DUOX1 methylation status was *n* = 148. DUOX1 methylation status was stratified into two groups by dividing the filtered data set at the median into a low methylation and a high methylation group. Both groups were plotted on a Kaplan–Meier curve and compared using the Gehan–Breslow–Wilcoxon test.

Cell Culture—Human PDAC cell lines MIA PaCa-2, PANC-1 and PDX-339 were cultured as previously described [7]. Human cell lines (MIA PaCa-2 and PANC-1) were purchased directly from the ATCC and were passaged for fewer than 6 months after receipt. No additional authentication was performed. The patient-derived cell line (PDX-339) was obtained from the Medical College of Wisconsin surgical oncology tissue bank [36,37]. Regardless of varying cell type and media components, all cells were treated with ascorbate in fresh 10% DMEM media for 1 h at 37 °C. Media was then replaced, ascorbate was removed, and cells were allowed to incubate for 48 h. Ascorbate came from a stock solution of 1 mol/L (pH 7) made under argon and stored with a tight-fitting stopper at 4 °C. Ascorbate concentration was verified at 265 nm, ε_265_ = 14,500 M^−1^ cm^−1^ [38]. To enhance rigor and reproducibility, final concentrations were calculated in units of moles-per-cell to account for variation in media, cell density, and cellular metabolism [39]. A 500 µM 5-azacytidine (AZC) (Sigma, A2385, St. Louis, MO, USA) solution was made in media and passed through a 0.22 µm filter. For in vitro use, a 100 µM 5-aza-2′-deoxycytidine (AZD) (Sigma, A3656) solution was made in distilled H_2_O, passed through a 0.22 µm filter, aliquoted and stored at −80 °C. For in vivo use, a separate 438 µM AZD (Tocris, 2624, Bristol, UK) solution was made in PBS (−/−) (Gibco, 14190144, Waltham, MA, USA), passed through a 0.22 µm filter, aliquoted and stored at −80 °C.

qRT-PCR-1 × 10^5^ cells were seeded for 5–7 days and then treated with 0.5–2 µM AZC for 5 days or 0.1–1 µM AZD for 3 days with fresh media and AZC or AZD replaced daily, with and without 10–20 pmol/cell P-AscH^−^ for 1 h. Following 5 days of AZC treatment or 3 days of AZD treatment and 48 h after P-AscH^−^, total cellular RNA was isolated using Trizol reagent (Invitrogen, 15596026, Waltham, MA, USA) in conjunction with the Direct-zol RNA Miniprep Kit (Zymo Research, Irvine, CA, USA) according to the manufacturer’s protocol. RNA was quantified using an ND1000 Nano-Drop spectrophotometer (ThermoFisher Scientific Co., Ltd., Waltham, MA, USA). cDNA was synthesized using a high-capacity cDNA archive kit (Applied Biosystems, Waltham, MA, USA). qRT-PCR assays were performed using 2× Power SYBR Green real-time master mix (Applied Biosystems, 4368702) under the following setup: 95 °C for 10 min, followed by 40 cycles of 95 °C for 15 s and 60 °C for 1 min (StepOne plus Sequence Detection System, Applied Biosystems). Primers were ordered from Integrated DNA Technology (DUOX1 gene ID: 53905, DUOX2 gene ID: 50506).

Primer sequences:

DUOX1 Forward-5′-GGATGCTGAGATCCTTCATCGAGA-3′

DUOX1 Reverse-5′-ACCTCCACCCCTTTGACACAGAG-3′

DUOX2 Forward-5′-TGTGTATGAGTGGCTGCCCAGC-3′

DUOX2 Reverse- 5′-ACTGCTCAGAGGCCACCACAAA-3′

Western Blotting—Protein samples were isolated and prepared in a phosphosafe buffer (EDM Millipore, 71296, Burlington, MA, USA) containing a protease inhibitor cocktail (Sigma). Protein sample cells were isolated in 1× RIPA buffer with a PhosSTOP phosphatase inhibitor cocktail (Sigma, 04906845001) and a complete Mini protease inhibitor cocktail (Sigma, 11836153001). Protein concentration was measured using a Bradford protein assay. Total protein (30–40 µg) was loaded on a 4–20% SDS-PAGE gradient gel (Bio-Rad). Membranes were blocked in 5% BSA in TBS-T. Primary antibodies included: DUOX1 (Santa Cruz Biotechnology, H-9, 1:1000, Dallas, TX, USA), DUOX2 (Santa Cruz Biotechnology, E-8, 1:1000, Dallas, TX, USA), DNMT1 (Santa Cruz Biotechnology, H-12, 1:1000), Tubulin (1:500–1000, University of Iowa Developmental Studies Hybridoma Bank, RRID: AB_528499), and GAPDH (Cell Signaling, D16H11, 1:1000). Appropriate horseradish peroxidase-linked secondary antibodies were used at a concentration of 1:20,000 to 1:50,000. Blots were visualized using SuperSignal West Pico PLUS substrate (Thermo Scientific, 34580) on X-ray film.

Measurement of Pro-oxidants—Bovine catalase (Sigma, C40) was utilized in the media at 100 mg/mL for 5 days along with 2 µM AZC replaced daily and immediately prior to P-AscH^−^ treatment (PDX-339, 1 mmol/L, which is equivalent to 10 pmol cell^−1^) for 1 h and fresh media was replaced. Cells were then allowed to proliferate for 48 h prior to the DCFH-DA (5-(and-6)-carboxy-2’,7’-dichlorodihydrofluorescein diacetate; Molecular Probes, C400) assay. Cells were incubated at 37 °C and protected from light in 15 mmol/L DCFH-DA for 30 min in PBS. They were then harvested and filtered for flow cytometry analysis. A CBD LSRII cytometer (BD Biosciences, San Jose, CA, USA) was used to measure DCFH-DA oxidation at 504/529 nm. Data were analyzed using FlowJo Software V10.

Clonogenic Survival—1 × 10^5^ cells were seeded 5–7 days prior to assay. Cells were treated alone or in combination with 0.5–2 µM AZC for 5 days, 0.1–0.3 µM AZD for 3 days, 10–20 pmol/cell P-AscH^−^ for 1 h, and bovine catalase (Sigma, C40) at 100 mg/mL. Following 5 days of AZC treatment or 3 days of AZD treatment and immediately after P-AscH^−^ treatment, cells were trypsinized with TrypLE Express (Gibco, 12604) to form a single-cell suspension and counted using a Countess II automated cell counter (Thermo Fisher) to determine the number of cells plated into each well. Cells were cultured for 10–14 days before being fixed and stained for analysis of surviving fraction. Colonies containing ≥50 cells were scored. The surviving fraction was defined as the number of colonies counted/number of cells seeded. Each experimental condition was normalized to its own control to determine a normalized surviving fraction. Each condition was carried out in triplicate, and experiments were performed at no less than *n* = 3.

In vivo Experiments—The animal protocols were reviewed and approved by the Animal Care and Use Committee of The University of Iowa. Thirty-day-old athymic nude mice (*Foxn*1^nu^) were obtained from Envigo and animals were allowed to acclimate in the unit for 1 week prior to any manipulation. MIA PaCa-2 cells (2 × 10^6^) were injected subcutaneously into each flank region of nude mice with a 1 mL tuberculin syringe equipped with a 25-gauge needle. Tumors grew to approximately 5 mm in diameter before experimental treatment began. Mice were divided into four treatment groups with equal tumor volume. Mice were treated once daily with I.P. saline (1 M), P-AscH^−^ (4 g/kg), AZD (1 g/kg), or a combination of P-AscH^−^ and AZD for 21 days. Tumor volume was measured twice weekly using handheld calipers. Mice were euthanized and tumors were harvested and processed for experimental analyses.

Immunofluorescent Staining—Mouse tumor tissue was fixed in paraformaldehyde and embedded in paraffin. Tissues were sectioned at 6–10 µm and placed on SuperFrost Plus slides and baked overnight at 42–45 °C. Slides were de-paraffinized and were further processed for immunohistochemistry. Immunofluorescent samples were subject to antigen unmasking protocol at 95 °C for 15 min, cooled to room temperature, washed with double distilled water, and allowed to dry. Samples were blocked with 5% normal goat serum prior to incubation with DUOX1 (1:50) and DNMT1 (1:10) primary antibodies in 1% normal goat serum at 4 °C for 24 h. Slides were washed 3 times in PBS for 5 min. Goat anti-mouse secondary (1:400) conjugated to FITC in 1% normal goat serum at 4 °C overnight. Slides were washed 3 times with PBS for 5 min. Nuclear staining was performed using Topoisomerase-3 (1:1000) for 15 min and samples were washed once with PBS. Coverslips were mounted on slides with immunofluorescence mounting medium and visualized using a Zeiss Confocal Microscope 40× oil objective. Quantification was performed using only DUOX1 or DNMT1 staining images. ImageJ was used to quantify the fluorescence for each image and values were normalized to each image’s nuclear content.

Statistical Methods—Data are presented as the mean ± SEM. For statistical analyses of two groups, unpaired 2-tailed Student’s *t*-tests were utilized. To study statistical differences between multiple comparisons, significance was determined using one-way ANOVA analysis with Tukey’s multiple-comparisons test. For survival analysis, the Gehan–Breslow–Wilcoxon test was used to compare overall survival. All analyses were performed in GraphPad Prism 9.0 (GraphPad Software, Inc., San Diego, CA, USA).

## 3. Results

**Figure 1:**
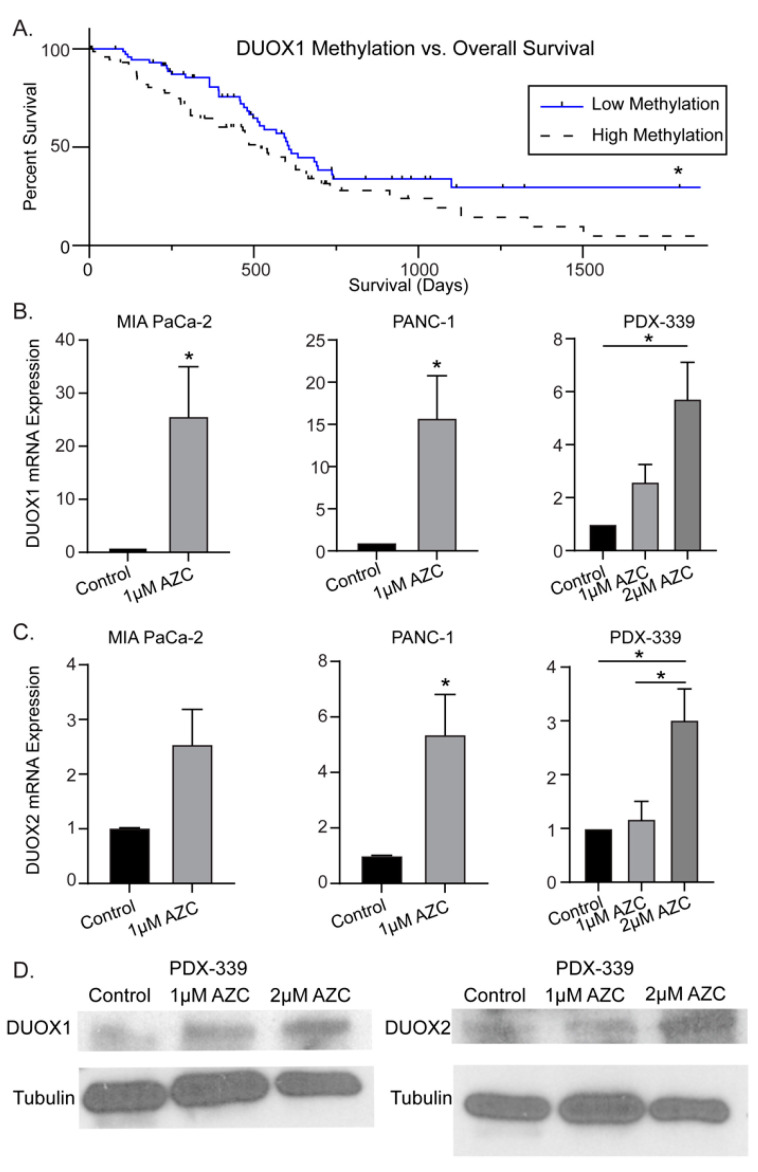
DUOX hypermethylation in PDAC. (**A**) DUOX1 methylation vs. overall survival in PDAC. The NIH Cancer Genome Atlas (TCGA) Pancreatic Cancer Database (PAAD) (*n* = 223) was accessed via the University of California Santa Cruz (UCSC) Xena Functional Genomics Explorer (*n* = 148; overall survival at 5 years 30% vs. 5%; * *p* = 0.03; Gehan–Breslow–Wilcoxon test). (**B**) DUOX1 mRNA expression is increased in a dose-dependent manner in the MIA PaCa-2, PANC-1, and PDX-339 PDAC cell lines after exposure to AZC (1–2 µM) for 5 days (means ± SEM; *n* = 3; * *p* < 0.05). (**C**) DUOX2 mRNA expression is increased in a dose-dependent manner in MIA PaCa-2, PANC-1, and PDX-339 PDAC cell lines after exposure to AZC (1–2 µM) for 5 days (means ± SEM; *n* = 3; * *p* < 0.05). (**D**) DUOX1 and DUOX2 immunoreactive protein was increased in PDX-339 cells after AZC (1–2 µM). Representative blots are shown.

DUOX1 methylation status correlates with overall survival in PDAC—Previous studies have examined the epigenetic landscape of PDAC, demonstrating silencing of tumor suppressor genes (p16, APC), DNA repair genes (MGMT), and normally expressed redox enzymes DUOX1 and DUOX2 [16,20,21,25,31]. Gibson et al. demonstrated that DUOX1 is silenced in PDAC, thus we hypothesized that increasing DUOX1 expression may improve overall survival in PDAC [31]. To determine if the differential expression of DUOX contributes to morbidity or mortality in PDAC, the Cancer Genome Atlas (TCGA) Pancreatic Cancer Database (PAAD) was utilized to evaluate the correlation between DUOX1 methylation status (as determined by Illumina Human Methylation 450 BeadChip) and overall survival [35]. In patients with PDAC, the cohort with low levels of methylation of the DUOX1 gene demonstrated significantly increased overall survival compared to patients with high levels of DUOX1 methylation, with approximately 30% of patients with low levels of DUOX1 methylation surviving at 5 years compared to 5% of patients with high levels of DUOX1 methylation (Figure 1A).

DNMT inhibitors increase DUOX1 and DUOX2 expression in PDAC—To evaluate DNMT inhibitor effectiveness in increasing DUOX expression, PDAC cell lines were treated with AZC (1–2 µM) and mRNA expression was determined by qRT-PCR. In vitro dosages of AZC were chosen based on previously observed data by Stresemann et al. demonstrating demethylation effects of AZC at 0.5–2 µM as well as clinical data suggesting an average plasma concentration of 3–11 µM in humans receiving standard dose AZC therapy [40,41]. AZC increased DUOX1 mRNA expression 25-fold in MIA PaCa-2 (25 ± 9), 15-fold in PANC-1 (15 ± 5), and 6-fold in PDX-339 cells (6 ± 1) (mean ± SEM, * *p* < 0.05) (Figure 1B). A similar trend is seen for DUOX2, with significant increases in expression following AZC treatment in all PDAC cell lines (Figure 1C). In addition, AZC increased the protein expression of both DUOX1 and DUOX2, demonstrating dose-dependent increases in protein expression in the PDX-339 cell line following AZC treatment (Figure 1D). These results suggest that DNMT inhibition may be a viable strategy to increase DUOX expression in PDAC.

**Figure 2:**
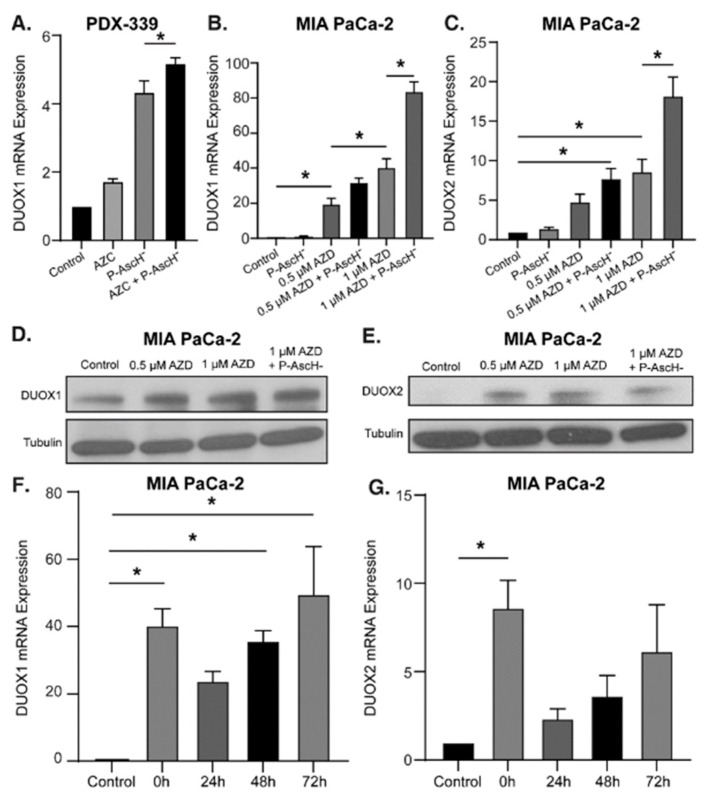
DNMT inhibitors with P-AscH^−^ increase DUOX expression in PDAC cell lines in a dose-dependent manner. Treatment with a DNMT inhibitor also produces sustained increases in DUOX expression. (**A**) DUOX1 mRNA expression is increased after exposure to AZC (2 µM) for 5 days ± P-AscH^−^ (20 pmol/cell) for 1 h in PDX-339 cells. The combination group demonstrated a significant increase in expression compared to either treatment group alone (means ± SEM; *n* = 3; * *p* < 0.05). (**B**) DUOX1 mRNA expression increased in a dose-dependent manner after exposure to AZD (0.5–1 µM) for 3 days in MIA PaCa-2 cells. The addition of P-AscH^−^ (10 pmol/cell) for 1 h produces a significant increase in expression compared to either treatment group alone (means ± SEM; *n* = 3; * *p* < 0.05). (**C**) DUOX2 mRNA expression increased in a similar manner with the same treatments. (**D**) DUOX1 immunoreactive protein increased in MIA-PaCa-2 cells with AZD (0.5–1 µM) for 3 days and P-AscH^−^ (10 pmol/cell) for 1 h. (**E**) DUOX2 immunoreactive protein increased with the same treatments. Representative blots are shown. (**F**) DUOX1 mRNA expression is increased for up to 72 h after exposure to AZD (1 µM). (**G**) DUOX2 mRNA expression is increased immediately following exposure to AZD (1 µM) (means ± SEM; *n* = 4; * *p* < 0.05 vs. control).

P-AscH^−^ combined with DNMT inhibitors further increases DUOX1 and DUOX2 expression—Previous studies have shown that P-AscH^−^ induces a prolonged increase in DUOX1 and DUOX2 expression as well as H_2_O_2_ production for up to 72 h in PDAC [31,39]. The hypothesized mechanism behind this increase is twofold. First, ascorbate acts as a cofactor for the TET family of enzymes, resulting in increased TET activity, DNA demethylation, and increased DUOX expression [13,14]. Second, while the DUOX1 enzyme produces H_2_O_2_, it is also activated by the presence of H_2_O_2_ supplied by both P-AscH^−^ and its own production, creating a positive feedback loop of enhanced and sustained H_2_O_2_ production [6,42]. To evaluate the combined effects of P-AscH^−^ and DNMT inhibitors on DUOX expression, PDAC cell lines were treated with AZC (2 µM) or AZD (0.5–1 µM) with and without P-AscH^−^ (10–20 pmol/cell). The addition of P-AscH^−^ to AZC-treated PDX-339 cells increased DUOX1 mRNA expression compared to either treatment alone (Figure 2A). There was a modest increase in expression observed for DUOX2 (Supplementary Figure S1), consistent with previous reports investigating DUOX2 expression after P-AscH^−^ [31]. Similar findings were seen in MIA PaCa-2 cells treated with AZD, demonstrating increases in DUOX1 (Figure 2B) and DUOX2 (Figure 2C) expression that are dose-dependent and further increased with the addition of P-AscH^−^. This increase in DUOX expression progressed with increasing doses of AZD up to 1 µM, where the addition of P-AscH^−^ increased DUOX1 mRNA expression (84 ± 5.5) compared to AZD alone (40 ± 5) (mean ± SEM, * *p* < 0.05). Treatment doses used in this experiment are easily achievable in humans, where plasma concentrations of 20 mM for P-AscH^−^ and up to 5 µM for AZD have been observed [9,43]. Western blot analysis demonstrates a similar trend for DUOX1 protein expression, with progressively increasing signal intensity with increasing doses of AZD and further increased intensity with the addition of P-AscH^−^ (Figure 2D). DUOX2 protein expression is also increased across all treatment groups compared to control (Figure 2E). These results reaffirm that epigenetic upregulation of these enzymes is possible in PDAC and that P-AscH^−^ and DNMT inhibitors may act synergistically to increase DUOX expression.

Previous studies have demonstrated that P-AscH^−^ induces a sustained increase in DUOX1 and DUOX2 expression in PDAC cells for up to 72 h [31]. To determine if DNMT inhibitors would induce a similar sustained increase in gene expression, MIA PaCa-2 cells were treated with 1 µM AZD for 3 d and mRNA expression was evaluated by qRT-PCR. AZD treatment increased DUOX1 expression for up to 72 h (Figure 2F) while there was increased DUOX2 expression observed immediately following treatment (Figure 2G). This sustained increase in DUOX1 expression induced by AZD could provide a mechanism to induce sustained H_2_O_2_ generation in PDAC between periods of treatment administration.

**Figure 3:**
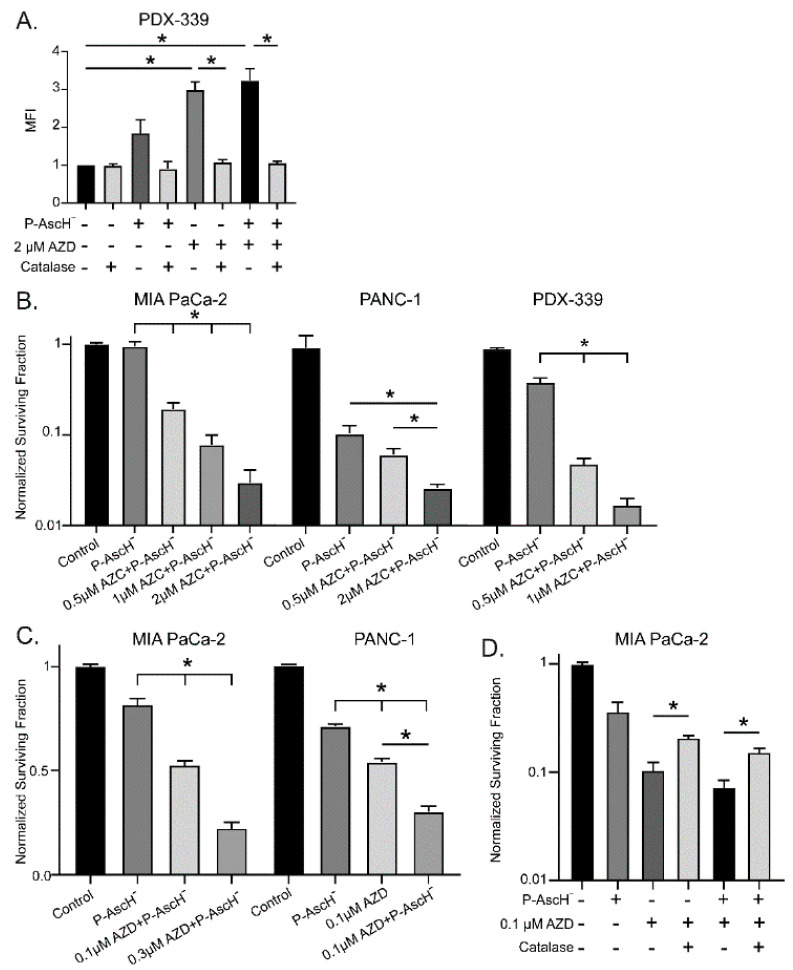
DNMT inhibitors and P-AscH^−^ generate dose-dependent, H_2_O_2_-dependent cytotoxicity. (**A**) Mean fluorescence intensity (MFI) is increased following exposure to AZC (2 µM) for 5 days and P-AscH^−^ (20 pmol/cell) for 1 h in PDX-339 cells. Pretreatment with bovine catalase (100 µg/mL) reverses this effect demonstrating that the oxidation of DCFH-DA is mediated by H_2_O_2_ (means ± SEM; *n* = 3; * *p* < 0.05). (**B**) MIA PaCa-2, PANC-1 and PDX-339 cells were treated with AZC (0.5–2 µM) for 5 days ± P-AscH^−^ (20 pmol/cell) for 1 h demonstrating decreases in clonogenic survival with the combination treatments (means ± SEM; *n* = 3; * *p* < 0.05). (**C**) MIA PaCa-2 and PANC-1 cells were treated with AZD ± P-AscH^−^ demonstrating decreases in clonogenic survival with the combination treatments (means ± SEM; *n* = 3; * *p* < 0.05). (**D**) Clonogenic survival in MIA PaCa-2 treated with AZD (0.1 µM), P-AscH^−^ (10 pmol/cell), and catalase (100 µg/mL). Catalase reverses the decrease in clonogenic survival supporting a H_2_O_2_ mechanism (means ± SEM; *n* = 3; * *p* < 0.01).

P-AscH^−^ and DNMT inhibitors increase hydrogen peroxide resulting in dose-dependent toxicity—To determine if this increase in DUOX expression translated to an increase in peroxide levels, intracellular reactive oxygen species were measured using a DCFH-DA oxidation assay. PDX-339 cells treated with 20 pmol/cell P-AscH^−^ for 1 h and 2 µM AZC for 5 d demonstrate significantly increased levels of DCFH-DA oxidation (Figure 3A). Pretreatment with catalase reverses this effect, demonstrating that H_2_O_2_ is the primary species oxidizing DCFH-DA following P-AscH^−^ and AZC treatment (Figure 3A). To evaluate the associated cytotoxic effects, clonogenic survival assays were performed following treatment with either 0.5–2 µM AZC or 0.1–0.3 µM AZD in combination with 10–20 pmol/cell P-AscH^−^. MIA PaCa-2, PANC-1, and PDX-339 cell lines all demonstrate that the addition of AZC to P-AscH^−^ significantly decreases cell survival in a dose-dependent manner compared to P-AscH^−^ alone (Figure 3B). Clonogenic survival studies were repeated in MIA PaCa-2 and PANC-1 cell lines using AZD. Again, PDAC cells demonstrate significantly decreased survival following the addition of AZD to P-AscH^−^ (Figure 3C). These studies demonstrate that P-AscH^−^ and DNMT inhibitors increase cytotoxic effects in PDAC cells when used in combination.

As we have demonstrated that DUOX expression and subsequent H_2_O_2_ production and cytotoxicity are all increased following P-AscH^−^ and DNMT inhibitor treatment, catalase was utilized to evaluate the degree to which H_2_O_2_ generation contributed to clonogenic survival. Clonogenic survival assays were repeated in MIA PaCa-2 cells treated with P-AscH^−^, AZD, and catalase. The decreased clonogenic survival of PDAC cells with AZD and P-AscH^−^ was reversed with catalase, suggesting a peroxide-mediated mechanism of toxicity following DNMT inhibitor treatment (Figure 3D).

**Figure 4:**
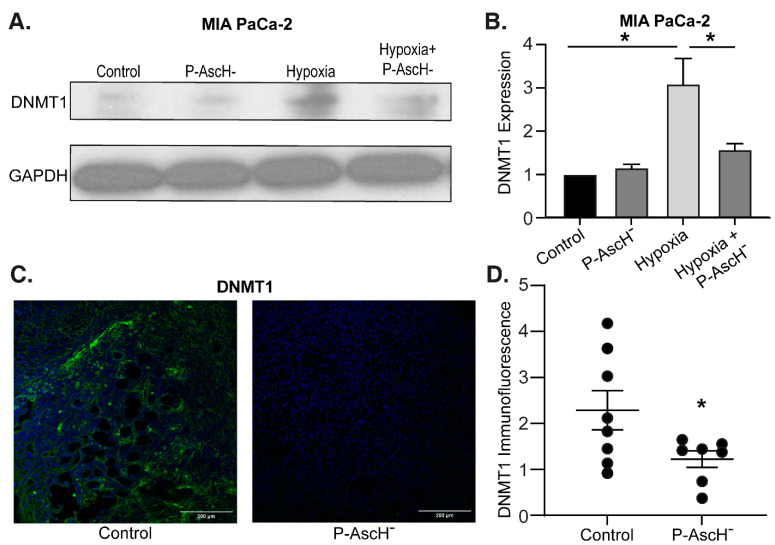
The hypoxia-induced increase in DNMT1 expression is decreased with P-AscH^−^. (**A**) DNMT1 immunoreactive protein was increased after exposure to 4% O_2_ for 6 h and decreased to baseline following exposure to P-AscH^−^ (10 pmol/cell). Representative blots are shown. (**B**) Quantification of densitometric evaluation of Western blots (mean ± SEM, values normalized to control; *n* = 3; * *p* < 0.05). (**C**) DNMT1 immunofluorescence staining was performed on xenograft tumor samples. Samples were visualized using a Zeiss Confocal Microscope 40× oil objective. Results show decreased DNMT1 immunofluorescence in the P-AscH^−^ treatment group compared to control. Green staining, DNMT1; blue staining, nuclear topoisomerase-3. Representative images are shown. (**D**) Quantification demonstrating MFI normalized to nuclear content (means ± SEM; *n* = 8; * *p* < 0.05).

Hypoxia-induced DNMT1 overexpression is decreased following P-AscH^−^ both in vitro and in vivo—Hypermethylation is commonly observed across multiple cancers, and DNMT overexpression has been suggested to increase tumorigenesis and worsen prognosis [20,21,22,23,24]. Furthermore, PDAC is known to exhibit hypoxic regions (partial oxygen pressure of 0–0.7%) relative to normal pancreatic tissue (3.2–12.3%) secondary to significant desmoplasia and fibrotic stroma [44]. To determine the effects of hypoxia on DNMT1 in PDAC cells, MIA PaCa-2 cells were exposed to 6 h of 4% oxygen with and without P-AscH^−^ treatment, and protein was isolated for Western blot analysis. Increased DNMT1 protein expression in hypoxia was reversed with P-AscH^−^ (Figure 4A). Quantification of the densitometry of multiple Western blots demonstrates that DNMT1 expression is significantly increased following 6 h of hypoxia and is significantly decreased following P-AscH^−^ treatment (Figure 4B).

Similar findings were seen in vivo. MIA PaCa-2 tumor xenografts in nude mice were allowed to grow to approximately 5 mm in diameter. At this time mice were divided into two groups and treated for 3 weeks with NaCl (controls, 4 g/kg 1 mol/L daily) or P-AscH^−^ (4 g/kg daily). After treatment, tumors were excised and processed for immunofluorescence. Compared to mice receiving saline, xenografts from mice treated with P-AscH^−^ demonstrated decreased DNMT1 immunofluorescence compared to the control group (Figure 4C). Quantification of the P-AscH^−^-induced decrease in DNMT1 immunofluorescence is demonstrated in Figure 4D, which shows a significant decrease in DNMT1 immunofluorescence in the P-AscH^−^-treated cohort. These results suggest that under the known hypoxic conditions of PDAC, the potential increase in DNMT expression may be decreased with P-AscH^−^, reducing DNA hypermethylation.

**Figure 5:**
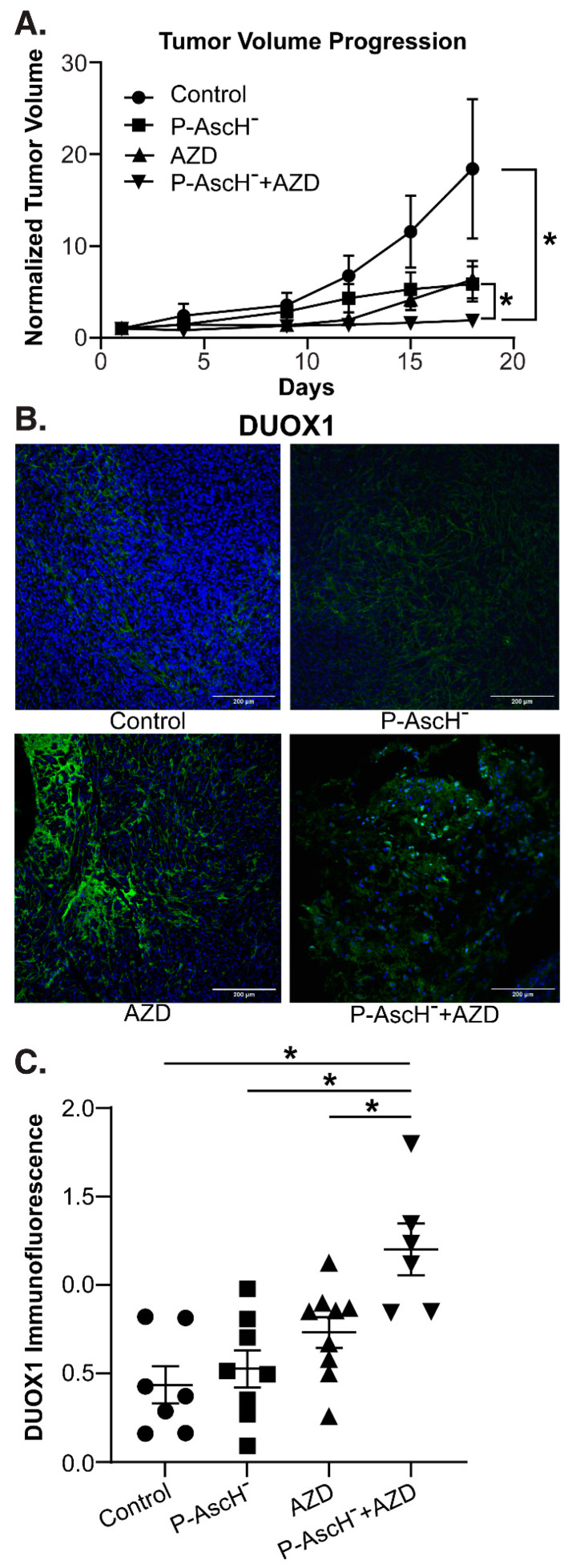
DNMT inhibitors combined with P-AscH^−^ decrease tumor volume and increase DUOX1 expression in vivo. Athymic nude mice with heterotopic MIA PaCa-2 xenografts were treated with intraperitoneal normal saline (4 g/kg, 1 M, daily), AZD (1 g/kg, three times weekly), P-AscH^−^ (4 g/kg, daily), or a combination of P-AscH^−^ and AZD. Mice were treated for 21 days and tumor volume was measured twice weekly. (**A**) Tumor growth was significantly inhibited in the combination AZD + P-AscH^−^ group compared to the control group and compared to either treatment group alone. Tumor volumes (mm^3^) were normalized to their starting volumes on treatment day 1 to avoid heterogeneity in the starting tumor volumes. Data represent average tumor volume over 18 d (means ± SEM; * *p* < 0.05). (**B**) DUOX1 immunofluorescence staining was performed on xenograft tumor samples. Samples were visualized using a Zeiss Confocal Microscope 40× oil objective. Results show increased DUOX1 immunofluorescence in the AZD treatment groups compared to control. Green staining, DUOX1; blue staining, nuclear topoisomerase-3. Representative images are shown. (**C**) Quantification demonstrating MFI normalized to nuclear content (means ± SEM; *n* = 7; * *p* < 0.05).

P-AscH^−^ and AZD combine to increase DUOX1 expression and decrease tumor growth in vivo—To determine the effects of P-AscH^−^ and AZD in vivo, separate groups of mice with established MIA PaCa-2 tumor xenografts were divided into four groups and treated for 3 weeks with NaCl (controls, 4 g/kg 1 mol/L daily), P-AscH^−^ (4 g/kg daily), AZD (1 g/kg, three times weekly), or a combination of P-AscH^−^ and AZD. Tumor volume was measured twice weekly. After treatment, the mice were euthanized, and tumors were excised and processed for immunofluorescence. The mice in the combination treatment group demonstrated significantly decreased tumor volume compared to the control group and compared to either treatment alone (Figure 5A). Tumor samples were also stained for DUOX1 and immunofluorescence was quantified. Tumors isolated from mice treated with both P-AscH^−^ and AZD demonstrated significantly increased DUOX1 immunofluorescence (Figure 5B) compared to the control group and to either treatment alone (Figure 5C). These in vivo studies demonstrate that the combination treatment of P-AscH^−^ and DNMT inhibitors such as AZD may offer a unique alternative or adjunct to traditional chemotherapy, with the epigenetic implications of a sustained increase in DUOX expression, decreased DNMT1 expression, and increased endogenous H_2_O_2_ production and subsequent H_2_O_2_-induced cytotoxicity.

## 4. Discussion

Even with advancements in chemotherapy regimens and radiation, median survival for PDAC is still extremely low, with a 5-year relative survival of just 11% across all stages and median survival of approximately 18 months for locally advanced disease [1,2,3,4]. P-AscH^−^ has shown promise as an adjunct to current chemo- and radiotherapy in recent phase I and II clinical trials [10,11,12]. Identifying therapies that can act synergistically with P-AscH^−^ would offer a novel approach to improve cancer-specific cytotoxicity. Our results demonstrate that P-AscH^−^ and DNMT inhibitors have at least additive and potentially synergistic effects on increasing cytotoxicity in pancreatic cancer cells.

Epigenetic modifications of tumor suppressor genes have been studied extensively. In PDAC specifically, the NADPH oxidase enzymes DUOX1 and DUOX2 are downregulated, but their expression is increased following exposure to H_2_O_2_ or P-AscH^−^ [31,42]. Available data suggest that DUOX1 is also often suppressed in hepatocellular carcinoma, a carcinoma of epithelial origin similar to PDAC [34,45]. In hepatocellular carcinoma, DUOX1 expression has been found to correlate with improved overall survival [45]. As seen in Figure 1A, we demonstrate the association of DUOX1 hypermethylation with decreased overall survival in PDAC. Thus, therapies that can act with P-AscH^−^ to increase DUOX expression and thereby increase downstream H_2_O_2_ production may benefit PDAC treatment.

Recent studies demonstrate that DNMT1 is overexpressed in PDAC, and its expression increases with progressing malignant potential, i.e., from pre-neoplastic lesions to invasive cancer [46]. This suggests that DNMT inhibitors may be effective adjuncts for PDAC treatment and offer an effective pathway to increase the expression of epigenetically silenced tumor suppressor genes, including DUOX. We demonstrate that commonly utilized DNMT inhibitors (5-azacytidine [AZC] and 5-aza-2′-deoxycytidine [AZD]) produce sustained increases in DUOX expression, leading to increased H_2_O_2_ production and H_2_O_2_-induced cytotoxicity. Our present study also demonstrates that modest concentrations of AZC (1 µM) and AZD (0.5–1 µM) produce significant increases in DUOX1 and DUOX2 expression across multiple PDAC cell lines compared to control [40,43]. These increases in DUOX expression are dose-dependent and significantly increased with the addition of P-AscH^−^, with DUOX1 mRNA expression increasing more than 80-fold following treatment with AZD and P-AscH^−^. Furthermore, the increase in DUOX1 expression is sustained for up to 72 h in the absence of treatment, which was also seen in vivo, where DUOX1 expression in the combination treatment group is increased compared to either treatment alone. The pathway for this large increase in enzyme expression is likely multifaceted and includes decreased genomic methylation secondary to decreased DNMT activity and increased degradation by DNMT inhibitors, decreased genomic methylation secondary to increased TET activity by P-AscH^−^, and the H_2_O_2_-induced increased expression and activity of DUOX enzymes from P-AscH^−^ and the endogenously produced H_2_O_2_ [6,13,14]. This robust pathway to increased DUOX expression may offer a consistent, sustained mechanism for increasing H_2_O_2_ production in PDAC.

Increased DUOX expression correlates with increased production of H_2_O_2_. The PDX-339 cell line was chosen for this experiment as PDX cell lines have been shown to retain native tumor heterogeneity in vitro and may more accurately reproduce clinical response [47]. Multiple previous studies have demonstrated the cancer-specific cytotoxic properties of H_2_O_2_ in PDAC [6,8]. Across all PDAC cell lines, we demonstrate significantly increased cytotoxicity and decreased clonogenic survival when DNMT inhibitors are combined with P-AscH^−^ compared with P-AscH^−^ alone, with surviving fractions less than 10% at conservative treatment doses. Similar to DUOX expression, these effects are dose-dependent. With the addition of catalase to the media prior to AZD or P-AscH^−^ treatment, the decreased clonogenic survival of PDAC cells was reversed, indicating a H_2_O_2_-mediated mechanism of cytotoxicity following DNMT inhibitor treatment. Although there is a complicated mechanism of carboxy-H_2_DCF-DA for its oxidation by H_2_O_2_ [48] we chose carboxy-H_2_DCF-DA and this approach because it is an indicator of intracellular levels of H_2_O_2_. Amplex red and related probes that use horseradish peroxidase provide information on extracellular H_2_O_2_, (i.e., H_2_O_2_ that may leak out of cells as well as any background H_2_O_2_ produced in the medium) have signals that can be very weak and often difficult to interpret because of poor signal-to-noise. Therefore, while the oxidation of carboxy-H_2_DCF to carboxy-DCF is complicated, it can be used to probe for changes in the flux of intracellular oxidants. The totality of our data points to increased levels of H_2_O_2_. These results are replicated in vivo, where the combination of AZD and P-AscH^−^ demonstrates inhibited tumor growth in a pancreatic cancer xenograft model. While P-AscH^−^ and DNMT inhibitors have been utilized independently in in vivo models of PDAC, this is the first study to demonstrate the combination leading to tumor growth inhibition [11,27,30,46].

Hypoxia is a well-known modulator of the PDAC microenvironment. Low oxygen content can impact enzyme function, cellular stability, DNA repair mechanisms, redox states, sensitivity to therapies, and gene expression [49,50]. We demonstrate that hypoxia increases DNMT1 protein expression in PDAC and that P-AscH^−^ reverses this effect. Similar results were seen in the in vivo tumor xenografts suggesting that hypoxia could be another factor leading to DNMT1 overexpression and hypermethylation in PDAC.

## 5. Conclusions

Treatments for PDAC must continue to evolve. Epigenetic alterations provide new opportunities for PDAC treatment. We have shown that sustained increases in DUOX expression can be achieved with DNMT inhibitors and that P-AscH^−^ enhances the cytotoxic and epigenetic effects of DNMT inhibitors through a peroxide-mediated mechanism. P-AscH^−^ decreases DNMT1 expression in hypoxia in vitro and in vivo, providing an additional mechanism to limit DNMT1 activity and DNA methylation. Finally, the combination of a DNMT inhibitor and P-AscH^−^ in vivo increases DUOX1 expression and decreases tumor volume compared to either treatment alone. Phase I and II clinical trials of DNMT inhibitors in PDAC are currently underway, where these therapies in combination with P-AscH^−^ may provide new avenues for PDAC treatment through an epigenetic mechanism.

## Data Availability

All data are contained within the manuscript.

## Supplementary Materials

The following supporting information can be downloaded at: https://www.mdpi.com/article/10.3390/antiox12091683/s1, Figure S1: DUOX2 mRNA expression is increased after exposure to AZC (2 µM) for 5 days and/or P-AscH^−^ (20 pmol/cell) for 1 hour in PDX-339 cells. The combination of AZC and P-AscH^−^ produces a similar increase in mRNA expression as AZC or P-AscH^−^ alone (means ± SEM, values normalized to control; *n* = 6; * *p* < 0.05 vs. control).
