# Supplementary material for: Pharmacologic Ascorbate and DNMT Inhibitors Increase DUOX Expression and Peroxide-Mediated Toxicity in Pancreatic Cancer
Source: Antioxidants (Basel). 2023 Aug 29;12(9):1683. doi: 10.3390/antiox12091683 (PMC10525653; doi:10.3390/antiox12091683)
Supplement: Supplementary file 1 [file antioxidants-12-01683-s001.zip › antioxidants-2531259-supplementary.pdf]

antioxidants

MDPI

Supplementary Materials:

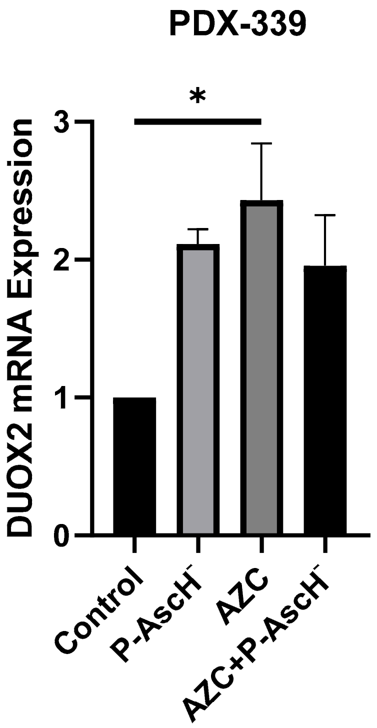

Figure S1. DUOX2 mRNA expression is increased after exposure to AZC (2 μM) for 5 days and/or P-AscH- (20 pmol/cell) for 1 hour in PDX-339 cells. The combination of AZC and P-AscH- produces a similar increase in mRNA expression as AZC or P-AscH- alone (means ± SEM, values normalized to control; $n = 6$; $*p < 0.05$ *vs*. control).
